# Improved resting state functional connectivity sensitivity and reproducibility using a multiband multi-echo acquisition

**DOI:** 10.1016/j.neuroimage.2020.117461

**Published:** 2020-10-16

**Authors:** Alexander D. Cohen, Baolian Yang, Brice Fernandez, Suchandrima Banerjee, Yang Wang

**Affiliations:** a Department of Radiology, Medical College of Wisconsin, 8701 Watertown Plank Road, Milwaukee, WI 53226, United States; b GE Healthcare, Waukesha, WI, United States; c GE Healthcare, Buc, France; d GE Healthcare, Menlo Park, CA United States

**Keywords:** Resting state functional MRI, Multiband, Multi-echo, Multi-echo independent component analysis, Functional connectivity density, Reproducibility

## Abstract

Recent advances in functional MRI techniques include multiband (MB) imaging and multi-echo (ME) imaging. In MB imaging multiple slices are acquired simultaneously leading to significant increases in temporal and spatial resolution. Multi-echo imaging enables multiple echoes to be acquired in one shot, where the ME images can be used to denoise the BOLD time series and increase BOLD sensitivity. In this study, resting state fMRI (rs-fMRI) data were collected using a combined MBME sequence and compared to an MB single echo sequence. In total, 29 subjects were imaged, and 18 of them returned within two weeks for repeat imaging. Participants underwent one MBME scan with three echoes and one MB scan with one echo. Both datasets were processed using standard denoising and advanced denoising. Advanced denoising included multi-echo independent component analysis (ME-ICA) for the MBME data and ICA-AROMA for the MB data. Resting state functional connectivity (RSFC) was evaluated using both selective seed-based and whole grey matter (GM) region-of-interest (ROI) based approaches. The reproducibility of connectivity metrics was also analyzed in the repeat subjects. In addition, functional connectivity density (FCD), a data-driven approach that counts the number of significant connections, both within a local cluster and globally, with each voxel was analyzed. Regardless of the standard or advanced denoising technique, all seed-based RSFC was significantly higher for MBME compared to MB. Much more GM ROI combinations showed significantly higher RSFC for MBME vs. MB. Reproducibility, evaluated using the dice coefficient was significantly higher for MBME relative to MB data. Finally, FCD was also higher for MBME vs. MB data. This study showed higher RSFC for MBME vs. MB data using selected seed-based, whole GM ROI-based, and data-driven approaches. Reproducibility found also higher for MBME data. Taken together, these results indicate that MBME is a promising technique for rs-fMRI.

## 1. Introduction

In resting state functional MRI (rs-fMRI), images are rapidly collected while a participant is not performing a specific task. Distinct brain networks, aka resting state networks (RSNs), can be extracted by analyzing the synchronization of the spontaneous signal throughout the brain. Recent advances in echo planar imaging (EPI) have greatly improved the estimation of RSNs. For example, in multiband (MB) imaging, multiple slices are acquired simultaneously allowing for increased spatial and/or temporal resolution (Feinberg et al., 2010; Moeller et al., 2010; Setsompop et al., 2012). Multiband imaging was further developed and refined by the Human Connectome Project (HCP) (Smith et al., 2013). However, MB imaging is also associated with decreased SNR due to g-factor effects caused by the unaliasing process and/or smaller voxel sizes (Smith et al., 2013). In multi-echo (ME) EPI imaging, images are acquired at multiple echo times (TEs) in a single shot and allow for increased fMRI sensitivity (Fernandez et al., 2017; Kundu et al., 2012; Poser and Norris, 2009; Poser et al., 2006; Posse et al., 1999). This technique, however, is also coupled with decreased temporal and spatial resolution due to the increased echo train length and the need for reasonable TEs.

Both MB and ME imaging have been applied to rs-fMRI. For example, one study found that using MB to achieve TRs less than one second resulted in an increase in peak functional sensitivity of 60% (Feinberg et al., 2010). Another study evaluated functional connectivity in 20 healthy volunteers using varying MB acceleration factors. They found using moderate acceleration factors (≤ 4) resulted in increased sensitivity in identifying common brain networks (Preibisch et al.,2015). Other studies have shown increased temporal resolution can lead to an improved ability to remove breathing and cardiac related physiological artifacts (Griffanti et al., 2014).

In ME imaging, there are several techniques to combine the echoes. Echoes can be combined using a simple summation where the signal from each echo is summed together (Posse et al., 1999). In the T2* -weighted method, the signal from each echo is weighted by their T2* value, on a voxelwise basis, which is estimated using a log-linear fit (Posse et al., 1999). Another option is the normalized T2* -weighted technique where the weights are normalized so they sum to one (Fernandez et al., 2017; Poser et al., 2006). Finally, echoes can be combined weighted by the calculated voxelwise contrast to noise (CNR) ratios (Poser et al., 2006). All of these methods have been shown to increase fMRI sensitivity. In addition, by acquiring images at both short and long TEs, ME imaging along with echo combination can mitigate signal loss in areas of the brain with susceptibility-related signal dropout and/or short T2* (Fernandez et al., 2017; Kundu et al., 2013; Posse et al., 1999).

Furthermore, ME data can be denoised using the multi-echo independent component analysis (ME-ICA) technique to automatically separate BOLD and non-BOLD signals (Kundu et al., 2013; Kundu et al., 2012). As reported previously, ICA is first used to identify signal components representing the most variance in the data. Then, these components get classified as either BOLD or non-BOLD based on whether or not their amplitudes are linearly dependent on TE, respectively. The non-BOLD components are then regressed from the data. In an rs-fMRI study, Kundu et al. found higher resting state functional connectivity (RSFC) using ME-ICA denoising compared to standard denoising techniques (Kundu et al., 2012). This effect was enhanced in sub-cortical – cortical connectivity. Additional ME rs-fMRI studies have shown higher signal to noise ratios (SNR) and specificity using ME-ICA (Kundu et al., 2013) and the ability to separate slow-BOLD from non-BOLD signal drifts (Evans et al., 2015). Another study found ME-ICA denoised functional connectivity matched task-based activation more closely than traditionally denoised ME data (Lombardo et al., 2016) Finally, ME-ICA has shown robustness to motion and a better ability to differentiate true BOLD signal from motion artifacts compared to single echo methods and multi-echo methods using other ICA-based denoising techniques (Dipasquale et al., 2017).

A very limited number of studies have combined the MB and ME techniques to evaluate fMRI. Olafsson et al. compared MBME and single band ME (SBME) acquisitions (Olafsson et al., 2015). They found using ME-ICA with MBME data identified significantly more BOLD components compared with SBME. They theorized this was due to an increased number of samples and an improved ability to filter out physiological artifacts due to the increased temporal resolution. However, they did not evaluate the effects of denoising on fMRI connectivity metrics. Another study showed improved identification of language lateralization using combined MBME data compared with a single echo from the same MBME acquisition (Amemiya et al., 2019). Boyacioglu et al. found higher spatial specificity using MBME compared with SBME at high field strengths (7T) (Boyacioglu et al., 2015). Finally, Cohen et al. showed higher connectivity strength and extent using an MBME pseudo-continuous arterial spin labeling (pCASL) sequence with and without ME-ICA compared to a single echo from the same sequence (Cohen et al., 2017).

While these studies provide a solid foundation for using MBME sequences for rs-fMRI, there remain unanswered questions. First, no study so far has evaluated the reproducibility of rs-fMRI metrics using MBME. In addition, the studies using MBME combined with ME-ICA to evaluate RSFC used a single echo from the same acquisition (Amemiya et al., 2019; Cohen et al., 2017). This does not consider the potential benefits of increased temporal resolution inherent in MB acquisitions.

In contrast to seed-based approaches, which measure RSFC with an *a priori selected* region of interest (ROI), functional connectivity density mapping (FCDM) is a data-driven way to assess patterns of RSFC (Cohen et al., 2018; Tomasi and Volkow, 2010, 2011). For FCDM, Pearson correlation is used to assess local functional connectivity density (lFCD) by counting the number of connections in a local cluster surrounding a voxel, and global functional connectivity density (gFCD) by counting the number of connections to a voxel with every other voxel in the brain. One recent study found higher sensitivity, specificity, and reproducibility of FCD using an MB acquisition compared to a conventional single band single echo acquisition (Cohen et al., 2018), but no study has evaluated FCDM using MBME in comparison with an MB sequence.

In this study, RSFC was evaluated using seed-based approaches and the data-driven FCD technique. Data were collected in a group of healthy volunteers using both an MBME sequence and an MB sequence with the increased temporal resolution, but the same spatial resolution. A subset of participants also returned within two weeks of their initial scan session. This allowed the reproducibility of RSFC and FCD metrics to be analyzed. It was hypothesized RSFC and FCD using MBME scans would be higher compared to MB. It was also hypothesized robust removal of non-BOLD signals from MBME scans would result in higher reproducibility for RSFC and FCD.

## 2. Methods

### 2.1. Subjects

All subjects received written informed consent prior to participation in this study. This study was approved by local Institutional Review Board and was conducted in accordance with the Declaration of Helsinki. In total, 29 healthy volunteer subjects (Mean Age = 28.0 Range 20 – 46, 9 Male, 20 Female) participated in this study. Among them, 18 returned within two weeks to repeat the study for a total of 47 imaging sessions. Subjects were instructed to refrain from caffeine and tobacco for six hours prior to imaging.

### 2.2. Imaging

Imaging was performed on a 3T scanner (Signa Premier, GE Healthcare, Waukesha, WI) with a body transmit coil and a 32-channel NOVA (Nova Medical, Wilmington, MA) receive head coil. Maximum gradient strength was 70mT/m and the maximum slew rate was 170 mT/m/ms). First, a 3D T1-weighted MPRAGE anatomical image was acquired with TR/TE = 2200/2.8ms, FOV = 24cm, matrix size = 512 × 512 × 256, slice thickness = 0.5mm, voxel size = 0.47 × 0.47 × 0.5mm, and FA = 8°. Each subject then underwent two rs-fMRI acquisitions, an MB scan and an MBME scan. The MB scan had the following parameters: TR/TE = 650/30ms, FOV = 24cm, matrix size = 80 × 80 with slice thickness = 3mm (3 × 3 × 3mm voxel size), 11 slices with multiband factor = 4 (44 total slices), FA = 60°, and partial Fourier factor = 0.85. The MBME scan had the following parameters: TR/TE = 900/11,30,49ms, FOV = 24cm, matrix size = 80 × 80 with slice thickness = 3mm (3 × 3 × 3mm voxel size), 11 slices with multiband factor = 4 (44 total slices), FA = 60°, and partial Fourier factor = 0.85. Both scans had an EPI readout with in-plane acceleration (R) = 2. Resting state scans lasted six minutes each resulting in 554 volumes for the MB scans and 400 volumes for the MBME scans. During the resting state scans, subjects were instructed to close their eyes, but remain awake, refrain from any motion and not think about anything in particular. band

### 2.3. Analysis

A combination of Freesurfer (Fischl, 2012), AFNI (Cox, 1996, 2012), FSL (Jenkinson et al., 2012), and Matlab (The Mathworks, R2018a) was used for the data analysis. The HCP minimal preprocessing pipeline (Glasser et al., 2013) was adopted and modified, as detailed below.

#### 2.3.1. Anatomical processing

Anatomical processing used the *PreFreeSurferPipeline.sh* scripts from the HCP pipeline. First, the anatomical image was ACPC aligned using *aff2rigid* in FSL. Next, a brain mask was created using FNIRT-based brain extraction. The MPRAGE image was linearly registered to MNI space using *flirt* in FSL with 12 degrees of freedom (Jenkinson et al., 2002). Then, *fnirt* in FSL was used to non-linearly refine the registration. A masked reference image in MNI space was inversely warped to native space using the transformations determined above and used to mask the MPRAGE image to extract the brain. Finally, the MPRAGE brain-only image was registered to MNI space using *flirt* with 12 degrees of freedom followed by *fnirt*.

In addition, a FreeSurfer analysis was run for all subjects using the *recon-all* command on the ACPC-aligned MPRAGE dataset. The purpose was to extract individual brain parcellations to use for the ROI-based connectivity analysis.

#### 2.3.2. Functional preprocessing

Two separate analyses were performed. First, the MB and MBME data were processed identically using “standard” denoising procedures. Second, the MB and MBME data were reprocessed using “advanced” denoising procedures, an ICA-based strategy for Automatic Removal of Motion Artifacts (ICA-AROMA) (Dipasquale et al., 2017; Pruim et al., 2015) and ME-ICA respectively. Details are described below.

For both the MB and MBME datasets, the first 10 seconds worth of volumes were discarded to allow the signal to reach equilibrium. Next, both the MB and MBME datasets were volume registered to the first volume using *mcflirt* in FSL and the six rigid-body motion parameters were output. For the MBME data, only the first echo was registered. Subsequent echoes were registered using the transformation matrices from the first echo. The three echoes were then combined using the T2* -weighted approach (Posse et al., 1999).

Standard denoising: The standard denoising pipeline consisted of first, registering both datasets to the ACPC-aligned MPRAGE image using *epi_reg* in FSL and then registering both datasets to MNI space using the anatomical transformations computed in Section 2.3.1. The data was then smoothed using a 4mm full width at half maximum (FWHM) Gaussian kernel and detrended with a third-order polynomial. Nuisance regressors, including the six rigid-body motion parameters, white matter, and CSF signal were regressed from the data and then the data was bandpass filtered with 0.01 < f < 0.1 Hz.

Advanced Denoising of MB: The advanced denoising pipeline for the MB data consisted of registration first to the ACPC-aligned MPRAGE image using *epi_reg* in FSL and then to MNI space using the anatomical transformations computed in section 2.3.1. The data was smoothed using a 4mm full width at half maximum (FWHM) Gaussian kernel and then denoised using an ICA-based strategy for Automatic Removal of Motion Artifacts (ICA-AROMA) (Dipasquale et al., 2017; Pruim et al., 2015). ICA-AROMA is a data-driven technique which removes components related to motion from the data. On average 27.7 +/− 10.0 components were regressed from the data using ICA-AROMA. Finally, the data was bandpass filtered with 0.01 < f < 0.1 Hz.

Advanced Denoising of MBME: The advanced denoising pipeline for the MBME data utilized ME-ICA and the open source python script tedana.py version 0.0.8 (https://tedana.readthedocs.io/en/latest/) (DuPre et al., 2019; Kundu et al., 2013; Kundu et al., 2012). This technique, described in detail elsewhere, classifies independent components as BOLD or non-BOLD based on whether or not their amplitudes are linearly dependent on TE, respectively (Kundu et al., 2013; Kundu et al., 2012; Olafsson et al., 2015). Non-BOLD components were regressed out of the combined ME data resulting in a denoised dataset. On average 24.8 +/− 5.6 components were regressed from the data using ME-ICA. The denoised MBME dataset was then registered to the ACPC-aligned MPRAGE image using *epi_reg* in FSL, and subsequently registered to MNI space using the anatomical transformations computed in section 2.3.1. Finally, the ME-ICA data was smoothed using a 4mm FWHM Gaussian kernel and bandpass filtered with 0.01 < f < 0.1 Hz.

##### TSNR:

The temporal signal to noise ratio (tSNR) was computed before the smoothing step by dividing the voxelwise mean signal across time by the voxelwise standard deviation of the time series. To account for differing numbers of time points between MBME and MB scans the tSNR was multiplied by $\sqrt{N_{TP}}$ where *N_TP_* is the number of time points (Smith et al., 2013).

#### 2.3.3. ROI based RSFC

**Fig. 1.**
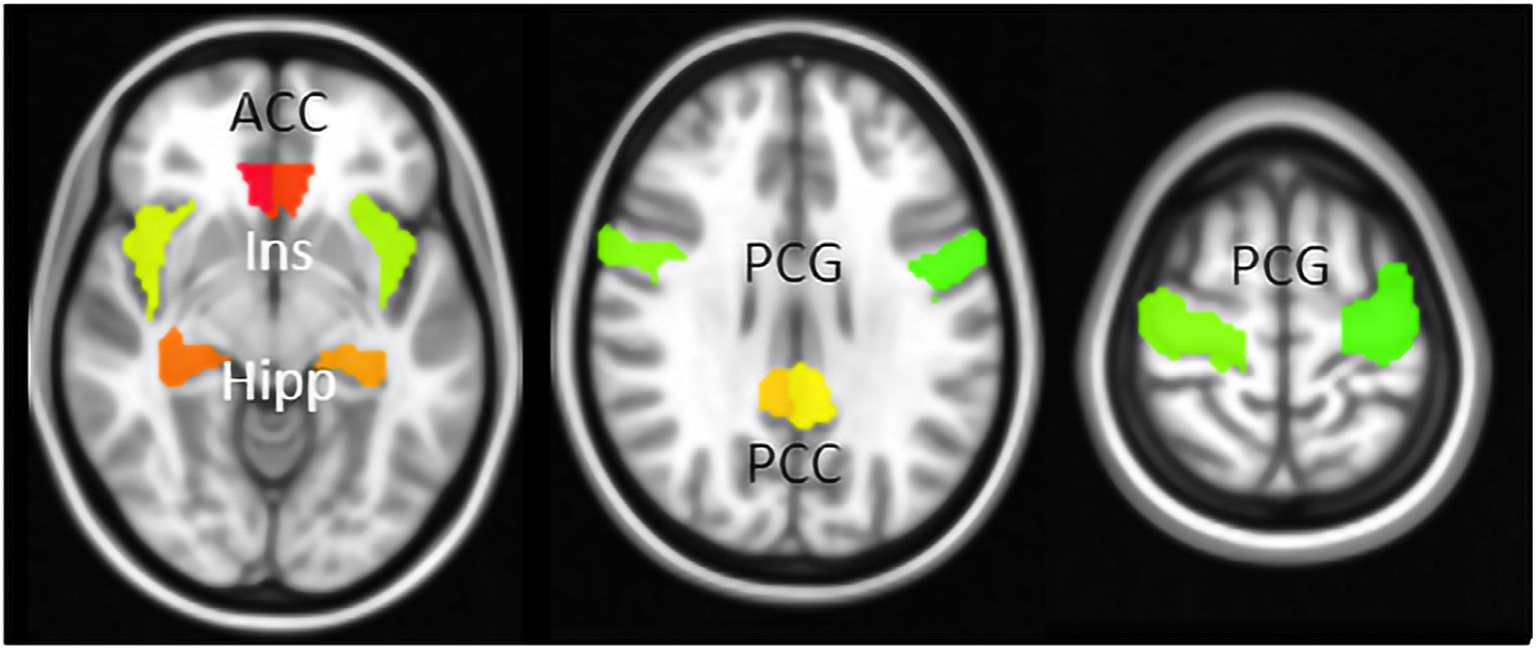
Selective seed-based ROIs. Ten seeds were chosen and include L/R anterior cingulate cortex (ACC), L/R insula (Ins), L/R hippocampus (Hipp), L/R posterior cingulate cortex (PCC), and L/R precentral gyrus (PCG). (Note: L=left, R=Right).

The preprocessed datasets were input into a selective seed-based ROI RSFC analysis. As described previously (Cohen et al., 2017), ten seeds were evaluated including L/R anterior cingulate cortex (ACC), L/R hippocampus (Hipp), L/R insula (Ins), L/R posterior cingulate cortex (PCC), and L/R precentral gyrus (PCG) (Fig. 1). Seeds were extracted from the automated anatomical atlas version 3 (AAL3) with the values 151, 152, 41, 42, 33, 34, 39, 40, 1, and 2, respectively (Rolls et al., 2020). The 10 ROIs used in the seed-based RSFC analysis are shown in Fig. 1. The mean signal time series within each seed was extracted and then correlated with every other voxel in the brain. For the standard denoised data, the number of degrees of freedom was reduced by 17 accounting for the 12 motion parameters, WM and CSF signal, and third order detrending. For the ME-ICA and ICA-AROMA denoised datasets, the number of degrees of freedom was adjusted on an individual subject basis to take into account the number of components regressed from the data. This only affected the significance thresholds for the individual datasets and did not factor into any of the group comparisons.

In addition, a whole grey matter (GM) ROI-based RSFC analysis was conducted using ROIs derived from the parcellations estimated by FreeSurfer on individual brains. For each subject, FreeSurfer parcellated the brain cortex into 148 ROIs. Besides, 16 subcortical ROIs from the AAL3 atlas were added to the analysis. The average signal from each of these ROIs was extracted from each dataset for each subject and correlated with the average signal from all other ROIs. This resulted in a 164 × 164 correlation matrix. The ROI-based correlation analysis was carried out using custom codes written in Matlab.

#### 2.3.4. Functional connectivity density

A FCD analysis was also conducted on all datasets. Both lFCD and gFCD were computed using *3dLFCD* and *3dDegreeCentrality* in AFNI, respectively. The number of voxels connected to a specific voxel was determined using Pearson correlation and a correlation threshold of r ≥ 0.6. Briefly, lFCD for a given voxel was defined as the size of the continuous cluster of spatially connected voxels that were all correlated with that voxel at r ≥ 0.6 (Cohen et al., 2018; Tomasi and Volkow, 2010, 2011). This procedure was repeated for every voxel in the brain (Kundu et al., 2012; Tomasi and Volkow, 2010, 2011). gFCD was computed by counting the number of voxels functionally connected to each voxel throughout the whole brain with r ≥ 0.6 (Tomasi and Volkow, 2011).

#### 2.3.5. Reproducibility

For the 18 subjects with two separate study sessions, reproducibility was assessed with the following metrics.

##### Dice coefficient:

The Dice coefficient (DC) provides a measure of the degree of overlap between two datasets. The DC ranges from 0 – 1 with DC = 1 meaning perfect overlap. The DC was computed for both the selective seed-based and whole-GM ROI-based RSFC analyses. For the selective seed-based analysis, first, for each subject and seed the RSFC maps were thresholded at r ≥ 0.3 for all datasets. The resulting maps were binarized with surviving voxels set to one. DC was then computed between time point one (TP1) and time point two (TP2) using Eq. (1). Here, OV_T1-T2_ is the number of overlapping voxels between TP1 and TP2, V_T1_ is the number of surviving voxels at TP1 and V_T2_ is the number of surviving voxels at TP2.


(1) $$DC=\frac{2\cdot OV_{T1-T2}}{V_{T1}+V_{T2}}$$

For the whole-GM ROI-based analysis, DC was calculated by first thresholding and binarizing the correlation matrices at four thresholds: r ≥ 0.3, 0.4, 0.5, and 0.6. Then, DC was calculated using Eq. (1) where OV_T1-T2_ is the number of overlapping ROI combinations between TP1 and TP2, V_T1_ is the number of surviving ROI combinations at TP1 and V_T2_ is the number of surviving ROI combinations at TP2. Because the correlation matrices are symmetric across the diagonal, only data below the diagonal was used for the DC calculations.

##### FCD reproducibility:

Reproducibility between TP1 and TP2 for lFCD and gFCD was estimated on a voxelwise basis using Eq. (2), which has previously been used in FCD analyses (Cohen et al., 2018; Tomasi et al., 2016) (Eq. (2)). Here FCD_T1_ is the FCD (either lFCD or gFCD) at TP1 and FCD_T2_ is the FCD (either lFCD or gFCD) at TP2. If values are ≥ 0, REP_FCD_ ranges from 0 – 1 with higher values being desirable. For example, if two values are the same, REP_FCD_ = 1. If the difference between two values is large, REP_FCD_ approaches 0. As opposed to percent change this metric controls for large outliers in the data. The lFCD and gFCD REP_FCD_ values were then averaged over GM for all datasets for each subject.


(2) $$REP_{FCD}=1-abs\left(\frac{FCD_{T1}-FCD_{T2}}{FCD_{T1}+FCD_{T2}}\right)$$

### 2.4. Statistics

#### 2.4.1. TSNR

Mean tSNR was extracted from GM using the FreeSurfer GM parcellations for each subject. The $tSNR\cdot \sqrt{N_{TP}}$ was compared between MB and MBME datasets using a paired t-test. A p-value < 0.05 was considered significant.

#### 2.4.2. Group analysis

For display purposes, selective seed-based RSFC maps were averaged across subjects for each of the 10 seeds for the all datasets and thresholded at r ≥ 0.4. A paired t-test was conducted between MB and MBME datasets using *3dttest*++ in AFNI. To account for the fact that some subjects had multiple scans, scan number was used as a covariate in the analysis. Maps were thresholded at P < 0.001 and the minimum cluster-size was estimated using *3dClustSim* in AFNI (Cox, 2019), so that the probability of a false positive cluster was controlled at *α* < 0.05.

Whole-GM ROI-based correlation matrices were also averaged across subjects for all datasets for visualization. A paired t-test was conducted between MBME and MB datasets for each ROI combination. The resulting t-score maps were thresholded at a False Discovery Rate (FDR) corrected q < 0.01. lFCD and gFCD maps were averaged across subjects for both all datasets. A paired t-test was then conducted between MB and MBME datasets for lFCD and gFCD using *3dttest*++ in AFNI. Scan number was once again used as a covariate in the analysis. As described above, t-score maps were thresholded at P < 0.001 and cluster-size corrected using *3dClustSim* in AFNI (Cox, 2019) at *α* < 0.05.

#### 2.4.3. Reproducibility

The DC was compared between MB and MBME datasets using a paired t-test for selective seed-based and whole-GM ROI-based RSFC. For the selective seed-based RSFC, DC was evaluated for each of the 10 seeds separately. A Bonferroni-corrected P < 0.05 was considered significant.

For lFCD and gFCD, the REP_FCD_ was averaged in GM and compared between MB and MBME datasets using a paired t-test.

### 2.5. Data availability statement

This study was funded by GE Healthcare. According to the research agreement between MCW and GE Healthcare for this study, data published in this paper will be available via a formal request to the authors and a data sharing agreement has to be signed by both parties.

## 3. Results

### 3.1. TSNR

#### 3.1.1. Standard denoising

The $tSNR\cdot \sqrt{N_{TP}}$ was significantly higher for the MBME datasets compared to the MB datasets (1802 +/− 147 vs. 1505 +/− 144 respectively, P << 0.001).

#### 3.1.2. Advanced denoising

The $tSNR\cdot \sqrt{N_{TP}}$ was significantly higher for the MBME datasets compared to the MB datasets (2248 +/− 197 vs. 1796 +/− 151 respectively, P << 0.001).

Furthermore, $tSNR\cdot \sqrt{N_{TP}}$ was significantly higher for MB data with ICA-AROMA denoising compared to MB data with standard denoising (1796 +/− 151 vs. 1505 +/− 144 respectively, P << 0.001). Similarly, $tSNR\cdot \sqrt{N_{TP}}$ was significantly higher for MBME data with ME-ICA denoising compared to MBME data with standard denoising (2248 +/− 197 vs. 1802 +/− 147 respectively, P << 0.001).

### 3.2. Selective seed-based RSFC

#### 3.2.1. Comparisons after standard denoising

**Fig. 2.**
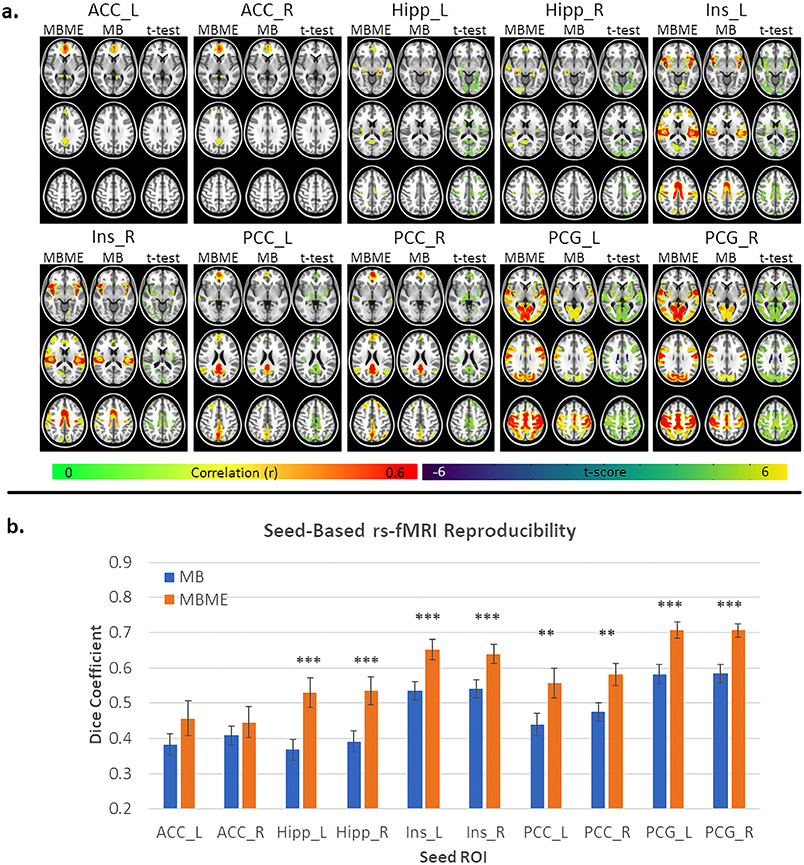
Seed-based functional connectivity and reproducibility for the standard denoising pipeline. Seed-based functional connectivity maps are shown in (a). For each seed, the first two columns are group averaged correlation maps for MBME and MB datasets respectively. The third column shows the results of a paired t-test between the MBME and MB data. Green/yellow colors indicate MBME > MB and blue colors indicate MBME < MB. Correlation maps were thresholded at r ≥ 0.4. T-test maps were thresholded at P < 0.001 and cluster size corrected at *α* < 0.05. For all seeds MBME correlation was higher than MB correlation. Mean DC, calculated using a correlation threshold of r ≥ 0.4, is shown in (b). The DC was significantly higher for MBME vs. MB for all seeds except L/R ACC. (Note: * = P < 0.05, * * = P < 0.01, * * * = P < 0.001. Abbreviations: MB = multiband single echo, MBME = multiband multi-echo).

Maps of the group mean correlation for the standard denoising analysis as well as paired t-tests between MB and MBME datasets are shown in Fig. 2a. For all seeds, the MBME data demonstrated higher group mean RSFC both in terms of the connectivity strength and the extent of the connectivity. This was confirmed by the paired t-test results which showed areas of significantly higher connectivity for MBME vs. MB scans for all seeds (p<0.001, cluster size corrected). The PCG seeds showed the largest areas of higher connectivity while not much difference was seen for the ACC seeds.

Dice coefficient results are shown in Fig. 2b. DC was significantly higher for MBME data vs. MB data for all seeds except the ACC seeds.

#### 3.2.2. Comparisons after advanced denoising

**Fig. 3.**
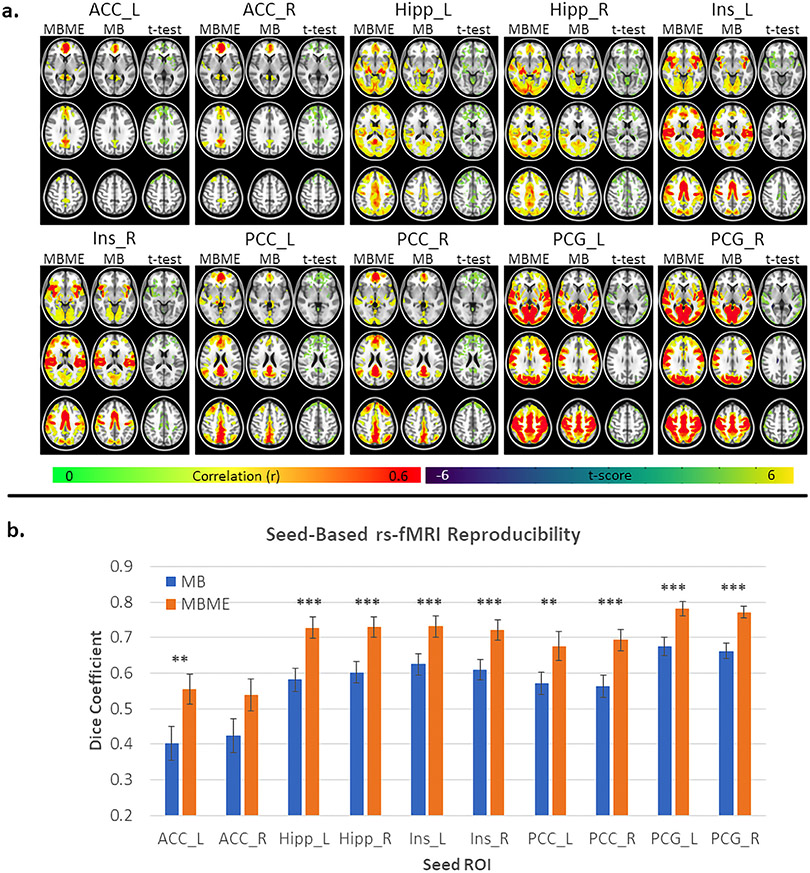
Seed-based functional connectivity and reproducibility for the advanced denoising pipeline. Seed-based functional connectivity maps are shown in (a). For each seed, the first two columns are group averaged correlation maps for MBME and MB datasets respectively. The third column shows the results of a paired t-test between the MBME and MB data. Green/yellow colors indicate MBME > MB and blue colors indicate MBME < MB. Correlation maps were thresholded at r ≥ 0.4. T-test maps were thresholded at P < 0.001 and cluster size corrected at *α* < 0.05. For all seeds MBME correlation was higher than MB correlation. Mean DC, calculated using a correlation threshold of r ≥ 0.4, is shown in (b). The DC was significantly higher for MBME vs. MB for all seeds except ACC_R. (Note: * = P < 0.05, * * = P < 0.01, * * * = P < 0.001. Abbreviations: MB = multiband single echo, MBME = multiband multi-echo).

Maps of the group mean correlation for the advanced denoising analysis, as well as paired t-tests between MB and MBME datasets are shown in Fig. 3a. For all seeds the MBME data had higher group mean RSFC both in terms of the connectivity strength and the extent of the connectivity. This was confirmed by the paired t-test results which showed areas of significantly higher connectivity for MBME vs. MB scans for all seeds (p<0.001, cluster size corrected). T-test results showed widespread higher connectivity for MBME vs. MB data for all seeds.

Dice coefficient results are shown in Fig. 3b. DC was significantly higher for MBME data vs. MB data for all seeds except the ACC_R seed.

### 3.3. Whole-GM ROI-based RSFC

#### 3.3.1. Comparisons after standard denoising

**Fig. 4.**
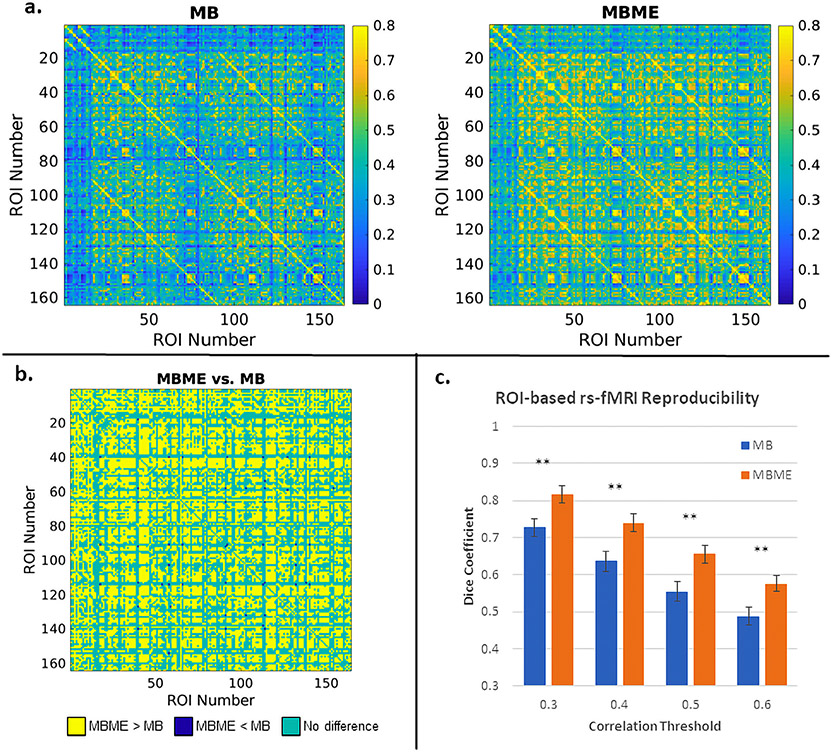
ROI-based functional connectivity and reproducibility for the standard denoising pipeline. (a) Mean correlation between ROI combinations across subjects for MB (left) and MBME (right) datasets. The first 16 ROIs are subcortical ROIs extracted using the AAL3 template. The remaining 148 ROIs are cortical ROIs extracted using each subject’s individual freesurfer parcellation. The mean correlation for the MBME data was higher than the MB data and also higher for cortical – cortical connections compared to subcortical – cortical connections for both datasets. (b) Paired t-test results for MBME vs. MB data. Yellow pixels indicate MBME > MB, green pixels indicate no difference and blue pixels indicate MBME < MB (FDR corrected q < 0.05). Overall, 52.7% of ROI combinations showed MBME > MB while 0.2% of ROI combinations showed MBME < MB. (c) The mean DC for four different correlation thresholds. For all thresholds, DC was significantly higher for MBME vs. MB datasets. (Note: * = P < 0.05, * * = P < 0.01, * * * = P < 0.001. Abbreviations: MB = multiband single echo, MBME = multiband multi-echo, DC = Dice coefficient).

Group mean correlation matrices for the standard denoising analysis are shown in Fig. 4a. Fig. 4b shows the results of the paired t-test between MBME and MB datasets (FDR corrected q < 0.05). Overall, 52.7% of all ROI combinations showed significantly higher correlation for MBME vs. MB datasets, while 0.2% of ROI combinations showed lower correlation for MBME vs. MB datasets.

Dice coefficient results are shown in Fig. 4c. DC was significantly higher for MBME vs. MB data for all thresholds.

#### 3.3.2. Comparisons after advanced denoising

**Fig. 5.**
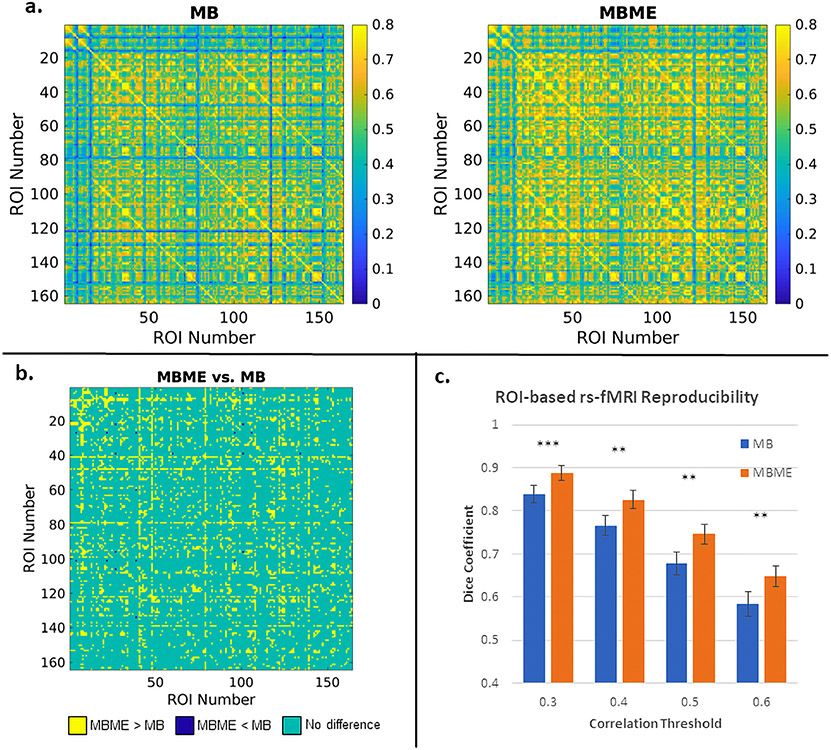
ROI-based functional connectivity and reproducibility for the advanced denoising pipeline. (a) Mean correlation between ROI combinations across subjects for MB (left) and MBME (right) datasets. The first 16 ROIs are subcortical ROIs extracted using the AAL3 template. The remaining 148 ROIs are cortical ROIs extracted using each subject’s individual freesurfer parcellation. The mean correlation for the MBME data was higher than the MB data and also higher for cortical – cortical connections compared to subcortical – cortical connections for both datasets. (b) Paired t-test results for MBME vs. MB data. Yellow pixels indicate MBME > MB, green pixels indicate no difference and blue pixels indicate MBME < MB (FDR corrected q < 0.05). Overall, 14.4% of ROI combinations showed MBME > MB, while only 0.04% of ROI combinations showed MBME < MB. (c) The mean DC for four different correlation thresholds. For all thresholds DC was significantly higher for MBME vs. MB datasets. (Note: * = P < 0.05, * * = P < 0.01, * * * = P < 0.001. Abbreviations: MB = multiband single echo, MBME = multiband multi-echo, DC = Dice coefficient).

Group mean correlation matrices for the advanced denoising analysis are shown in Fig. 5a. Fig. 5b shows the results of the paired t-test. Overall, 14.4% of all ROI combinations showed significantly higher correlation for MBME vs. MB datasets, while only 0.04% of ROI combinations showed lower correlation for MBME vs. MB datasets.

Dice coefficient results are shown in Fig. 5c. DC was significantly higher for MBME vs. MB data for all thresholds.

### 3.4. Functional connectivity density

#### 3.4.1. Comparisons after standard denoising

**Fig. 6.**
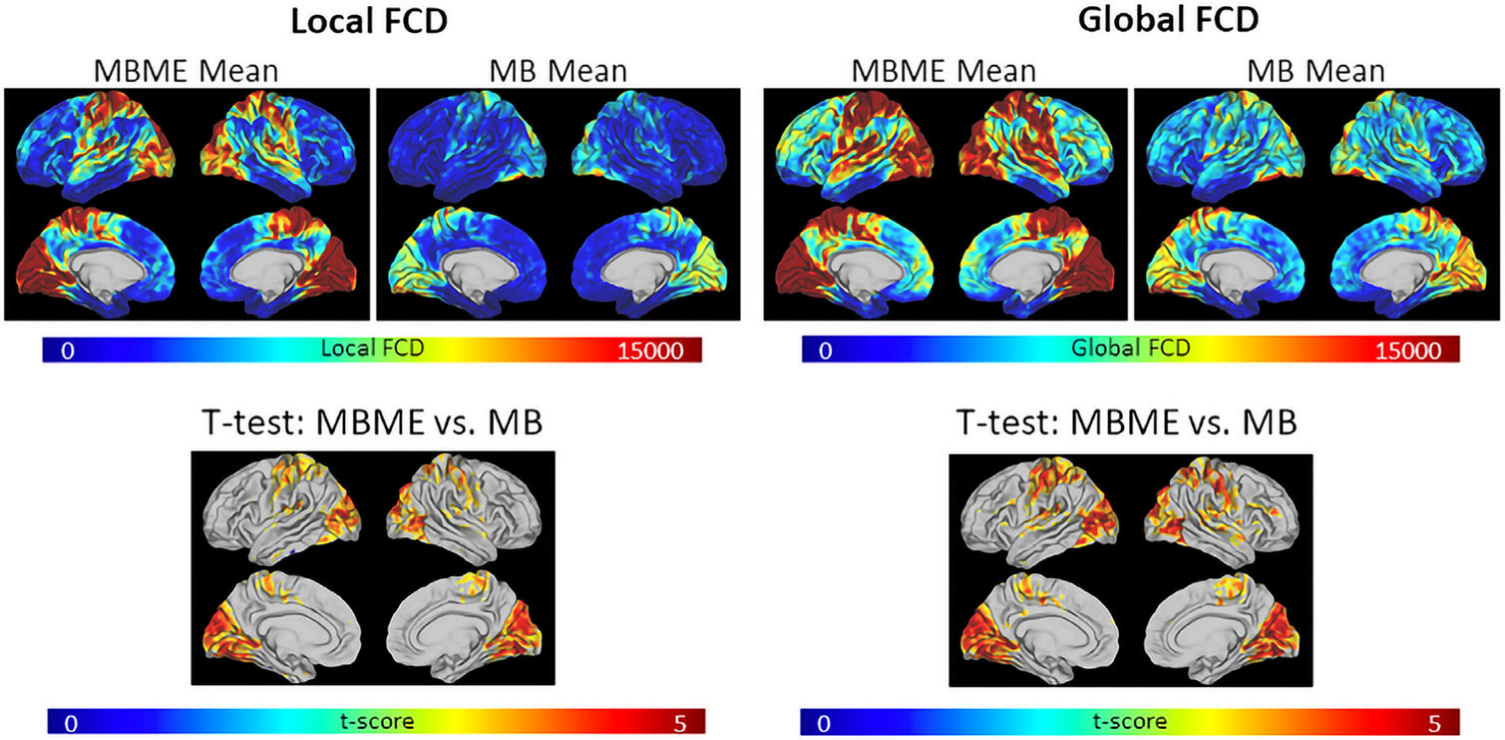
Maps of FCD and FCD reproducibility for the standard denoising pipeline. (a) Mean lFCD (left panel) and gFCD (right panel) are shown for MBME and MB datasets. Results of a paired t-test between MBME and MB datasets (bottom) are also shown. Both mean lFCD and mean gFCD were higher for MBME vs. MB. The t-test results showed significantly higher lFCD and gFCD in the in several regions, mainly in parietal, temporal and occipital cortices, for MBME vs. MB data (p < 0.001 and cluster size corrected at *α* < 0.05). No region showed significantly higher FCD in MB relative to MBME. (b) Reproducibility of lFCD and gFCD. Reproducibility was significantly higher for MB vs. MBME for lFCD. (Note: FCD = Fucntional Connectivity Density; lFCD = local FCD; gFCD = global FCD; MB = multiband single echo, MBME = multiband multi-echo).

Fig. 6 shows group mean lFCD and gFCD maps for for MB and MBME data after the standard denoising as well as t-test results comparing the MB and MBME datasets. Both MB and MBME datasets exhibited areas of heightened FCD in the visual and motor cortices as well as the default mode network. The paired t-test results for both lFCD and gFCD showed significantly higher FCD for MBME vs. MB in several regions, mainly in parietal, temporal and occipital cortices. No region showed significantly higher FCD in MB compared to MBME.

REP_lFCD_ was significantly higher for MB vs. MBME data (0.582 ± 0.073 vs. 0.537 ± 0.073 respectively, p = 0.012) while no significant difference in the REP_gFCD_ was seen between MB and MBME data (0.522 ± 0.082 vs. 0.526 ± 0.065 respectively).

#### 3.4.2. Comparisons after advanced denoising

**Fig. 7.**
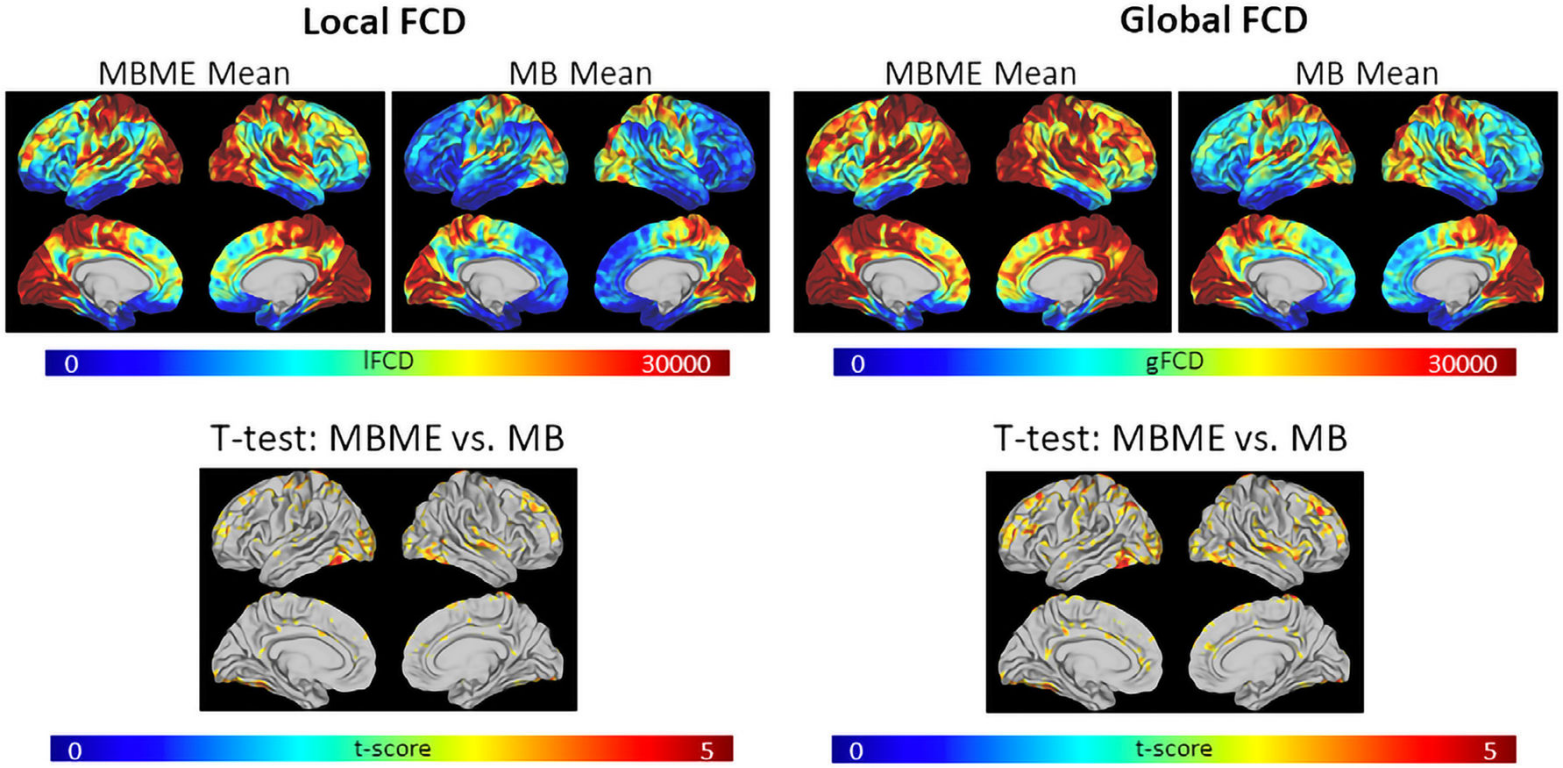
Maps of FCD and FCD reproducibility for the advanced denoising pipeline. (a) Mean lFCD (left panel) and gFCD (right panel) are shown for MBME and MB datasets. Results of a paired t-test between MBME and MB datasets (bottom) are also shown. Both mean lFCD and mean gFCD were higher for MBME vs. MB. The t-test results showed significantly higher lFCD and gFCD mainly in the frontal and temporal cortices as well as parietal areas for MBME vs. MB data (p < 0.001 and cluster size corrected at *α* < 0.05). No region showed significantly higher FCD in MB compared to MBME. (b) Reproducibility of lFCD and gFCD. Reproducibility was significantly higher for MBME vs. MB for gFCD. (Note: FCD = Fucntional Connectivity Density; lFCD = local FCD; gFCD = global FCD; MB = multiband single echo, MBME = multiband multi-echo).

Fig. 7 shows group mean lFCD and gFCD maps for MB and MBME data after the advanced denoising analysis as well as t-test results comparing the MB and MBME datasets. Both MB and MBME datasets exhibited areas of relatively high FCD in the visual and motor cortices as well as the default mode network. The paired t-test results for both lFCD and gFCD showed significantly higher FCD for MBME vs. MB mainly in the frontal and temporal cortices as well as parietal areas. No region showed significantly higher FCD in MB compared to MBME.

No difference in REP_lFCd_ was seen between MBME and MB datasets (0.486 ± 0.100 vs. 0.493 ± 0.048065 respectively) while REP_gFCD_ was higher for MBME compared to MB data (0.515 ± 0.090 vs. 0.468 ± 0.049065 respectively, p = 0.027).

## 4. Discussion

In this study, several measures of RSFC, including selective seed-based, whole-GM ROI-based, and data-driven FCD, were compared between MBME and MB acquisitions using standard or advanced denoising techniques. Connectivity strength and extent were higher for MBME scans compared to MB for all metrics. In addition, for most connectivity measures, reproducibility was higher for MBME scans relative to MB scans in subjects with repeated scans.

One advantage of ME scans is the ability to acquire a short echo time (<15ms). This allows BOLD contrast to be maximized in voxels with short T2* as signal from the first echo will be weighted more heavily in the T2* -weighted echo combination scheme. The collection of short TE images also increases signal in areas of susceptibility induced signal dropout mainly due to the increase in signal at short echo times. Another advantage of ME scans is that, in addition to being able to estimate T2* and combine echoes using T2* -weighting, ME-ICA can be used to denoise the data (Kundu et al., 2013; Kundu et al., 2012). This study showed a significantly higher tSNR for MBME data following ME-ICA denoising compared to MB following ICA-AROMA denoising. Previous studies have shown a comparatively small increase in tSNR can result in a large reduction in the number of time points necessary to detect significance (Murphy et al., 2007). Therefore, statistical power is greatly increased and the number of time points necessary to detect significance is reduced using ME-ICA. To illustrate this, MBME data was compared to MB data where the TR was reduced from 900ms to 650ms resulting in an increase in the number of timepoints from 400 for MBME to 554 for MB. Despite the increase in the number of volumes, MBME connectivity was still higher than MB. ME-ICA denoised MBME data was also more reproducible than the MB data for all connectivity metrics. ME-ICA removes artifactual non-BOLD signals from the data including R2* effects, motion artifacts, and physiological noise (Kundu et al., 2013; Kundu et al., 2012; Kundu et al., 2017). Thus, variations in these signals between scan sessions are mitigated automatically and result in a more stable signal over time.

To provide a fairer comparison of MBME and MB sequences two separate analyses were performed. In the first analysis, the two datasets were denoised with an identical standard approach. Connectivity and repeatability metrics were compared between these sequences. In the second analysis, the MBME and MB datasets were denoised with state of the art ICA-based denoising methods: ME-ICA for the MBME data and ICA-AROMA for the MB data. Of note, ICA-AROMA was chosen as the denoising strategy over other ICA-based denoising techniques such as FIX because ICA-AROMA has been shown to outperform FIX in previous studies (Dipasquale et al., 2017). Also, FIX requires a separate training dataset where signal and noise components are manually determined. Ideally, the training dataset should match the test dataset. For this study, there were not enough subjects to create our own FIX training dataset. The tSNR for the ICA-AROMA denoised MB data was significantly higher compared to the MB data with standard denoising. Regardless of the denoising technique, the MBME data outperformed the MB data showing higher connectivity strength and extent, higher FCD, and higher dice coefficients.

Comparing the standard and advanced denoised datasets directly, the advanced denoising approach derived higher connectivity strength and extent, higher FCD, and higher dice coefficients compared to their counterparts with standard denoising. Interestingly, differences between MBME and MB datasets were more pronounced for the standard denoising compared to advanced denoising. This can be seen for the ROI-based connectivity where 52% of voxels showed a significantly higher correlation for the MBME data vs. MB data compared to 14% for the advanced denoised data. This supports the notion that ICA-AROMA induced significant improvements in signal quality for the MB data (Dipasquale et al., 2017; Pruim et al., 2015).

The L/R hippocampus seed ROIs were chosen to evaluate subcortical – cortical connectivity. Kundu et al. showed more widespread hippocampal connectivity with ME-ICA compared to without and specifically showed a higher correlation with motor and sensory regions (Kundu et al., 2012). A comparable trend was seen for the insula seeds. Cohen et al. performed a similar connectivity analysis (Cohen et al., 2017). They also found widespread connectivity using the hippocampus and insula seeds following ME-ICA, which was significantly higher compared to ME data without ME-ICA and single echo data. Of note, the ME data without ME-ICA had higher connectivity compared to the SE data. Our results are in accord with the results from these two papers. Significantly higher correlation was seen for the MBME data compared to the MB data for the hippocampus seeds specifically in the motor and visual areas. Kundu et al. credit these improvements to the ability of ME-ICA to robustly identify the origin of each component by using spatial and TE information and then remove non-BOLD and/or noise components from the data (Kundu et al., 2012). Similar trends in subcortical – cortical connectivity were observed for the whole-GM ROI-based RSFC analysis. While subcortical – cortical connectivity was less than cortical – cortical connectivity for both MB and MBME datasets, subcortical – cortical connectivity was higher for the MBME data compared to MB data. This is quantified in Figs. 4 and 5 with many subcortical – cortical ROI combinations showing significantly higher correlation for MBME vs. MB datasets.

One thing to note is the widespread connectivity for most of the seeds for the MBME data following ME-ICA. As a result, a relatively high correlation threshold (r = 0.4) was used for visualization of the networks in Figs. 2 and 3. This is in line with prior studies using ME-ICA (Cohen et al., 2017; Kundu et al., 2012) where, for some seeds, significant connectivity was observed over most of the brain. This trend was also seen for the whole-GM ROI-based connectivity analysis. Kundu et al. noted this and developed a new seed-based connectivity analysis method based on ME-ICA termed ME independent coefficients regression (ME-ICR) (Kundu et al., 2015). The ME-ICR connectivity is determined using the intervoxel correlation of BOLD ICA coefficients. Using this technique, the RSFC is not as affected by global correlations, which may cause the widespread connectivity seen after ME-ICA. In this study, a one to one comparison between MBME and MB acquisitions was desired. Therefore, ME-ICR was not used.

The Dice coefficient was used to evaluate reproducibility. The DC measures the degree of overlap between two measures and, as a result, requires a threshold to be set. The DC changes with threshold and has been shown to decrease with the increased threshold. For example, one study examined the DC between task and rs-fMRI at different thresholds and found decreasing DC with the increasing threshold (Branco et al., 2016). Another study also analyzed the concordance between task and ICA-based rs-fMRI brain networks and found similar trends (Yahyavi-Firouz-Abadi et al., 2017). For this study, four correlation thresholds ranging from r = 0.3 – 0.6 were chosen to calculate the DC for both the seed-based (results not shown for r = 0.4, r = 0.5, and r = 0.6) and ROI-based analyses. While DC did decrease with increasing threshold, the DC for MBME data was found significantly higher than MB data for all thresholds.

FCD mapping was developed by Tomasi and Volkow (Tomasi and Volkow, 2010) to measure the number of local and global connections with each voxel in the brain. FCD requires the *a priori* selection of a correlation threshold. Here, based on the results of the selective seed-based and whole-GM ROI-based analyses, as well as previous studies (Tomasi et al., 2016; Tomasi and Volkow, 2010, 2011), a relatively high threshold of r = 0.6 was chosen. Tomasi and Volkow found a threshold of R < 0.4 led to an increased number of false positives and a threshold of R > 0.7 led to lower sensitivity and reduced dynamic range (Tomasi and Volkow, 2010). They also chose a threshold of r = 0.6. Cohen et al. applied FCDM to MB data and found higher reproducibility, sensitivity, and specificity for MB compared to SB data (Cohen et al., 2018). Here, it was shown these techniques can be also applied to a ME analysis and that MBME data with ME-ICA denoising increases lFCD and gFCD. These findings could have important implications, as FCD has been used in several applications. For example, FCD has been detected to be different between men and women (Tomasi and Volkow, 2012). In addition, FCD changes were found to be associated with depressive symptoms in aging male adults (Lan et al., 2015). Abnormal FCD has also been associated with non-epileptic (Ding et al., 2014) and epileptic seizures (Li et al., 2019), Parkinson’s disease (Zhang et al., 2015), and schizophrenia (Zhuo et al., 2014).

Despite the benefits of an MBME acquisition, limitations of this technique exist that require consideration. First, the additional echoes cause TR to be increased. Thus, all else equal, MB acquisitions can be collected faster than MBME acquisitions. Second, in order to obtain reasonable echo times, in-plane acceleration must be used. This reduces SNR and can increase anti-aliasing artifacts. In this study an in-plane acceleration of 2 was used. MB acquisitions don’t require in-plane acceleration. Thus, the MB factor can be increased without much g-factor related SNR loss resulting in a further decrease in TR. MB scans have the potential to be collected with TRs less than the cardiac frequency allowing for it to be directly sampled (Griffanti et al., 2014). Finally, because of the long echo-train length required for MBME, MBME acquisitions are not compatible with very high spatial resolutions. To provide an equal comparison between MBME and MB, the spatial resolution was matched for both acquisitions in this study. In practice, a tradeoff can be made for MB data between TR and voxel size. If high spatial resolution data is required, MBME may not be an option; however, advances in gradient technology, including strength and slew rate may alleviate this issue (Foo et al., 2018).

Additional limitations for this study include the fact that only young, healthy volunteers were enrolled. MBME with ME-ICA denoising should still have a beneficial impact in patient populations. In fact, in patient populations, where physiological variations and motion related noise may be increased, ME-ICA should have an even greater impact. Additional comparisons should be further studied in patient populations.

In conclusion, this study demonstrated higher selected seed-based and whole-GM ROI-based RSFC and FCD for MBME compared to MB data, regardless of denoising approaches. The reproducibility RSFC was also higher when using the MBME technique. Taken together, these results show MBME is a promising technique for rs-fMRI.

## Abbreviations:

rs-fMRI: resting state functional magnetic resonance imaging
RSFC: resting state functional connectivity
HCP: Human Connectome Project
MB: multiband
ME: multi-echo
EPI: Echo planar imaging
TE: echo time
BOLD: blood oxygenation level dependent
ME-ICA: multi-echo independent component analysis
CNR: contrast to noise ratio
SNR: signal to noise ratio
SBME: single band multi-echo
MBME: multiband multi-echo
ROI: region of interest
FCD: functional connectivity density
FCDM: functional connectivity density mapping
TR: repetition time
FOV: field of view
FA: flip angle
FIX: FMRIB’s ICA-based X-noiseifier
FWHM: full width at half maximum
tSNR: temporal signal to noise ratio
ACC: anterior cingulate cortex
Hipp: hippocampus
Ins: Insula
PCC: posterior cingulate cortex
PCG: precentral gyrus
AAL3: anatomical atlas version 3
DC: Dice coefficient
MPD: modified percent difference
lFCD: local functional connectivity density
gFCD: global functional connectitivy density
GM: gray matter
WM: white matter
pCASL: pseudo-continuous arterial spin labeling
TP: time point
ME-ICR: multi-echo independent coefficients regression
